# CLAS-Net: A study on cross-lingual intelligent sentiment analysis model fusing semantic alignment

**DOI:** 10.1371/journal.pone.0342342

**Published:** 2026-02-11

**Authors:** Jia-Qi Wang

**Affiliations:** School of Fine Arts and Design, Leshan Normal University, Le Shan, Si Chuan, China; University of the Chinese Academy of Sciences, CHINA

## Abstract

In the context of the deep integration of globalization and digitalization, the cross-lingual dissemination of news and public opinion information has become an increasingly significant challenge. This study proposes a novel cross-lingual sentiment analysis framework, CLAS-Net, designed to address the bottlenecks of current public opinion analysis systems in multilingual scenarios. The framework combines the cross-lingual contrastive learning capabilities of XLM-RoBERTa with the precise sentiment feature extraction ability of BiLSTM-Attention, enabling efficient analysis of multilingual public opinion. In monolingual tasks for English and Portuguese, CLAS-Net achieves accuracies of 92% and 89%, respectively, representing a 29 percentage point improvement compared to baseline models. In more challenging multilingual settings, CLAS-Net maintains a high accuracy of 83%, a 29 percentage point improvement over the baseline model. CLAS-Net (Cross-Lingual Alignment Sentiment Network) demonstrates strong adaptability and practical value when processing real-world social media and news data, providing reliable technical support for cross-lingual public opinion monitoring and analysis in the global context.

## 1 Introduction

### 1.1 Background

The deep integration of globalization and digitalization is reshaping the landscape of information dissemination. According to the DataReportal Global Digital Report, there are over 4.95 billion active internet users worldwide, with 87.9% of them accessing news and sharing information through social media. The widespread adoption of mobile internet has further accelerated this trend, with mobile-based news consumption accounting for 76.3% of the overall news access methods [1]. In this context, cross-lingual information dissemination has become an irreversible global phenomenon.

However, linguistic diversity presents significant challenges to global information sharing. According to the Ethnologue Language Statistics Report, there are 7,164 languages spoken globally, making cross-lingual information understanding and sentiment analysis extremely complex. Data from Ani Petrosyan’s statistics further highlights the urgency of this challenge: by October 2024, the global number of internet users had reached 5.52 billion, accounting for 67.5% of the global population. Among them, 5.22 billion were social media users, comprising 63.8% of the global population [2]. This indicates that more language communities are joining the global information network, generating vast amounts of multilingual content.

**Fig 1:**
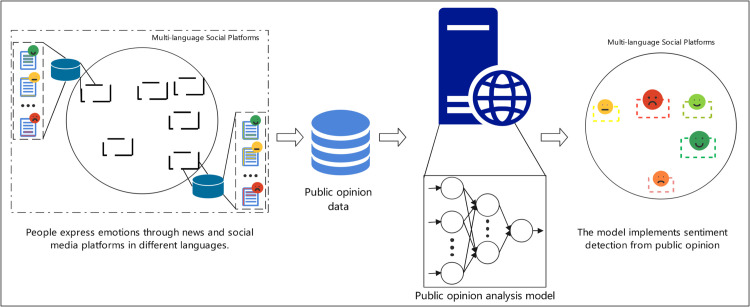
Public opinion sentiment analysis.

In an era characterized by linguistic diversity and rapid information dissemination, an effective global public opinion analysis framework becomes particularly important [3]. As shown in Fig 1, current information dissemination exhibits multilingual and multi-channel characteristics: global users express their views and emotions through various news media and social platforms, transcending traditional language and cultural boundaries.

In this context, accurately understanding and analyzing public opinion across different languages is crucial. However, existing public opinion analysis systems face significant limitations in multilingual scenarios [4]. These challenges stem from semantic differences and cultural features between languages that complicate sentiment understanding, insufficient real-time analysis capabilities for emerging news events, and poor performance when handling low-resource languages. These issues limit timely public opinion monitoring and crisis warning capabilities.

Therefore, establishing a system that can accurately understand multilingual sentiments and monitor global public opinion in real-time is of great importance. This study proposes a novel cross-lingual sentiment analysis framework, CLAS-Net (Cross-Lingual Alignment Sentiment Network). The framework combines the cross-lingual contrastive learning capabilities of XLM-RoBERTa with the precise sentiment feature extraction ability of BiLSTM-Attention, enabling efficient analysis of multilingual public opinion. Through the contrastive learning mechanism, the system aligns the semantic spaces of different languages, captures shared features, and uses deep learning techniques for accurate sentiment analysis.

### 1.2 Related work

Existing approaches in public opinion monitoring systems and multilingual sentiment analysis techniques have made substantial progress. However, critical bottlenecks and limitations persist in current methods. A systematic examination of these approaches reveals important insights about their strengths and weaknesses, providing the foundation for identifying specific challenges that the CLAS-Net framework is designed to overcome.

In the field of public opinion monitoring, surveillance technologies have continuously evolved with the development of social media. Li et al. [5] proposed a cross-dimensional mining model based on fuzzy association rules, which performed well in medium-scale data analysis but showed significant efficiency decline when processing large-scale real-time data. Karamouzas et al. [6] developed an automated public opinion monitoring mechanism based on Twitter messages, though its semantic understanding accuracy in multilingual environments still needs improvement. While Song et al. [7] proposed a parallel incremental association rule mining framework that enhanced mining efficiency, it lacks deep understanding of multilingual semantics. These studies indicate that existing public opinion monitoring systems face notable bottlenecks in multilingual environments, necessitating more effective language feature extraction methods.

While these public opinion monitoring systems represent important progress in surveillance methodologies, they share a critical limitation that fundamentally constrains their effectiveness in global contexts: inadequate handling of multilingual content. The studies referenced above primarily focus on efficiency improvements and mining techniques for monolingual data, yet fail to address the semantic understanding challenges inherent in cross-lingual sentiment analysis [8]. This gap becomes increasingly critical given that modern public opinion dissemination—as highlighted in the background section—is inherently multilingual [9]. The bottleneck lies not merely in processing speed or data scale, but in the fundamental inability to establish semantic correspondence between sentiment expressions across different languages [10]. To effectively monitor global public opinion, public opinion systems must be enhanced with robust multilingual sentiment analysis capabilities that can bridge these linguistic boundaries while maintaining analytical accuracy.

Multilingual sentiment analysis technology, as a key solution to the aforementioned problems, has achieved breakthrough progress in recent years. He et al. [11] proposed the LGCF model, which achieved significant performance improvements on Chinese and English benchmark datasets, though cross-lingual semantic alignment still requires optimization. Vargas et al. [12] developed the EurOpi technique enabling cross-lingual opinion comparison, but it relies heavily on pre-built knowledge bases. The MSA-GCN model proposed by Mercha et al. [13] exhibits high computational complexity when processing large-scale corpora.

In summary, existing research primarily faces two key challenges: semantic alignment and feature representation, demonstrating the urgency of exploring more effective cross-lingual feature extraction methods. The research analysis in Table 1 further confirms the necessity of this research direction.

**Table 1:** Analysis of cross-lingual sentiment detection literature.

Authors	Application Scenario	Research Content	Potential Limitations
Hassan et al. [14]	Cross-lingual Sentiment Detection	Explored three cross-lingual approaches: multilingual models, training data translation, and auto-labeled parallel corpora	Insufficient utilization of contrastive learning for semantic space alignment, potentially affecting cross-lingual transfer effectiveness
An et al. [15]	Cross-lingual Sarcasm Detection	Zero-shot cross-lingual transfer method based on prompt learning, combining data augmentation and contrastive learning	Focus limited to English-to-Chinese transfer, lacking validation on other language pairs
Bhattacharya et al. [16]	Gender-related Sentiment Detection	Utilized deep learning models for cross-corpus sentiment and gender identification	Lacks specific mechanisms for low-resource languages, limited model generalization capability
Miok et al. [17]	Parliamentary Speech Analysis	Multi-aspect analysis of parliamentary speeches from six countries, including sentiment and viewpoint analysis	Neglects real-time requirements, lacking rapid response capability for emergent events
Acheampong et al. [18]	Text Sentiment Detection	Survey of BERT-based models in sentiment detection	Primarily focused on English context, insufficient attention to multilingual scenarios
Zehra et al. [19]	Cross-corpus Speech Emotion Recognition	Employed ensemble learning methods for multilingual speech emotion recognition	Insufficient utilization of deep semantic features, potentially affecting complex emotion recognition accuracy
Ghosh et al. [20]	Code-mixed Text Analysis	Multi-task learning for sentiment and emotion in Hindi-English mixed text	Limited to bilingual mixed scenarios, not extended to multi-language mixing cases
Qian et al. [21]	Cultural Understanding	Utilized contextual learning and masked language modeling for cross-cultural analysis	Lacks dedicated sentiment alignment mechanisms, potentially affecting cross-cultural sentiment understanding
Spiesberger et al. [22]	Speech Abuse Detection	Used acoustic features to detect abusive speech in Indian languages	Lack of text feature integration, potentially affecting detection accuracy in complex scenarios
Ahmad et al. [23]	Crisis Response Sentiment Analysis	Constructed multilingual sentiment detection framework for disaster domain	Limited data scale covering only English and Hindi, restricted generalization ability

The literature analysis presented in Table 1 synthesizes representative cross-lingual and multilingual sentiment analysis approaches, revealing a consistent pattern of limitations across existing work. While diverse research directions have been explored—ranging from zero-shot cross-lingual transfer methods to deep learning approaches for code-mixed text—the table highlights a fundamental gap: existing work typically addresses individual aspects of the multilingual sentiment analysis challenge rather than providing unified, comprehensive solutions. Specifically, Hassan et al., An et al., and Bhattacharya et al. demonstrate that current approaches often excel in specific language pairs or restricted scenarios but lack generalization mechanisms for diverse multilingual contexts. Ahmad et al.’s framework, though addressing multilingual sentiment detection, remains limited to English and Hindi, constraining its applicability. These observations underscore that the research community has not yet achieved a balanced framework capable of simultaneously addressing semantic alignment, robust feature extraction across languages, and practical deployment across low-resource languages.

Among the most prominent existing multilingual models—mBERT, XLM, and their variants—significant limitations emerge when examined in light of these real-world requirements [24]. mBERT demonstrates notable performance imbalances across languages because its pre-training relies on uneven language representation, with some languages receiving substantially more training data than others, resulting in significantly degraded accuracy for low-resource languages. Additionally, the frozen embeddings in mBERT after pre-training limit its adaptability to task-specific sentiment expressions, particularly when emotional nuances vary across linguistic contexts [25]. XLM-based approaches address some multilingual challenges through cross-lingual pre-training objectives, yet they rely primarily on static masked language modeling without task-specific alignment mechanisms [26]. This design limitation results in suboptimal semantic correspondence in downstream sentiment analysis tasks, where capturing precise emotional equivalence across languages is essential [27]. Traditional BiLSTM-based methods, when applied to multilingual scenarios, typically process each language independently without establishing explicit cross-lingual feature bridges, failing to capture the shared semantic structures that enable effective multilingual understanding.

These limitations indicate that existing models often focus on specific language pairs, lack dedicated mechanisms for low-resource languages, or fail to effectively integrate cross-lingual learning with sentiment-specific feature extraction [28]. To address these identified gaps, CLAS-Net is designed as a unified framework that overcomes the limitations of existing cross-lingual sentiment analysis approaches [29]. At its core, CLAS-Net leverages XLM-RoBERTa’s pre-trained cross-lingual contrastive learning capabilities to establish more robust semantic correspondences between languages, moving beyond the basic translation or word-level alignment strategies employed by existing methods. The contrastive learning mechanism explicitly aligns sentiment expressions across languages in a shared semantic space, directly addressing mBERT’s frozen representation and XLM’s lack of task-specific alignment. Furthermore, by integrating BiLSTM-Attention, the model effectively captures long-distance emotional dependencies and dynamically emphasizes sentiment-bearing expressions across languages, thereby overcoming the limitations of transformer-only designs that fail to adequately handle the sequential nature of sentiment expressions and of independent monolingual processing that neglects cross-lingual connections. The key innovation lies in the synergistic combination of XLM-RoBERTa’s cross-lingual representation learning with BiLSTM-Attention’s sentiment-specific processing capabilities. This architectural design creates a unified approach that enables CLAS-Net to maintain high sentiment classification accuracy across diverse language contexts while simultaneously preserving multilingual consistency—a balance that most existing approaches fail to achieve. Through this integrated framework, CLAS-Net effectively bridges linguistic boundaries while delivering superior sentiment analysis performance.

### 1.3 Our contributions

Building on the identified gaps in existing work, our main contributions are as follows:

1. This study proposes CLAS-Net, a novel cross-lingual sentiment analysis architecture that integrates improved XLM-RoBERTa and BiLSTM-Attention mechanisms, directly addressing the semantic alignment and feature extraction limitations prevalent in existing multilingual models such as mBERT and XLM-based approaches. Unlike existing methods that prioritize either semantic alignment or sentiment-specific processing but not both, CLAS-Net achieves a balanced integration of cross-lingual representation learning and sentiment-specific feature refinement. Experimental results demonstrate that this architecture achieves 92% and 89% accuracy in English and Portuguese sentiment analysis tasks respectively, while maintaining 83% accuracy in multilingual scenarios—performance levels that exceed those reported in comparative studies including Hassan et al., An et al., and Bhattacharya et al.’s work.
2. The study introduces an efficient cross-lingual feature extraction method that overcomes the low-resource language limitations identified in Table 1 (Bhattacharya et al., Ahmad et al.) by leveraging XLM-RoBERTa’s balanced multilingual pre-training foundation combined with an improved attention mechanism. This approach enables the model to capture sentiment expression features across different languages with unprecedented consistency, avoiding the performance degradation observed in existing methods when applied to under-represented languages. The model demonstrates strong fine-grained classification capability, achieving 34.0% accuracy when handling challenging neutral sentiment categories—a significant improvement over the implicit treatment of neutral sentiment in many existing approaches.
3. A comprehensive multilingual sentiment analysis evaluation framework is presented, including systematic experimental protocols for both monolingual and multilingual scenarios. The model’s performance across different linguistic environments is assessed through multiple dimensions, including accuracy metrics, loss curves, and confusion matrices.

## 2 Research methodology

This section presents the formal problem formulation for multilingual sentiment analysis and introduces the CLAS-Net architecture. The methodology comprises three interconnected components that work synergistically to address the challenge of cross-lingual sentiment analysis. The first addresses the problem through mathematical formalization, the second through XLM-RoBERTa based semantic feature extraction, and the third through BiLSTM-Attention based sentiment classification. The integration of these components is detailed in the algorithm overview.

### 2.1 Problem description

In the context of global information dissemination, the core challenges in multilingual sentiment analysis can be formally described through the following mathematical formulation. First, we define the multilingual text collection in the global information space:

$$𝒯=\{T_{l_{1}},T_{l_{2}},...,T_{l_{n}}\}$$ (1)

where $T_{l_{i}}$ represents the text collection in language *l*_*i*_, and *n* denotes the number of languages to be monitored. The real-time information flow within each text collection can be expressed as:

$$T_{l_{i}}(t)=\{x_{1}^{l_{i}}(t),x_{2}^{l_{i}}(t),...,x_{m_{i}}^{l_{i}}(t)\}$$ (2)

where *t* represents the time point, and $x_{j}^{l_{i}}(t)$ denotes the *j*th piece of information in language *l*_*i*_ at time *t*. Considering the variations in sentiment expression due to cultural differences, we define the cross-lingual sentiment space:

$$𝒮=\{{𝒮}_{l_{1}},{𝒮}_{l_{2}},...,{𝒮}_{l_{n}}\}$$ (3)

where ${𝒮}_{l_{i}}$ represents the sentiment expression space of language *l*_*i*_. In practical monitoring, information propagation exhibits time delays, expressed as:

$$\Delta t_{i,j}=t_{receive}^{j}-t_{publish}^{i}$$ (4)

where $t_{publish}^{i}$ is the publication time in the source language and $t_{receive}^{j}$ is the reception time in the target language. For emergent events, the information diffusion intensity can be described as:

$$I(t)=\sum_{i=1}^{n}\sum_{x\in T_{l_{i}}(t)}v(x)\cdot r(x)$$ (5)

where *v*(*x*) represents the information propagation velocity and *r*(*x*) denotes the information influence range. Considering the imbalance in language resources, we define language resource richness:

$$R(l_{i})=\frac{\left|T_{l_{i}}\right|}{\sum_{j=1}^{n}|T_{l_{j}}|}\cdot \log(\frac{\left|D_{l_{i}}\right|}{\left|D_{min}\right|})$$ (6)

where $\left|T_{l_{i}}\right|$ represents the text volume in language *l*_*i*_, $\left|D_{l_{i}}\right|$ denotes the dictionary size of that language, and |*D*_*min*_| is the minimum dictionary size among all languages. To address cultural differences in sentiment expression, we introduce a cultural distance metric:

$$d_{c}(l_{i},l_{j})=\sqrt{\sum_{k=1}^{K}(c_{k}^{i}-c_{k}^{j}{)}^{2}}$$ (7)

where $c_{k}^{i}$ and $c_{k}^{j}$ represent the scores of two languages on the *k*th cultural dimension, and *K* is the total number of cultural dimensions.

**Problem 1.** *Based on the above formalization, the core problem of multilingual sentiment analysis can be stated as: Given the multilingual information space 𝒯, how to achieve accurate cross-lingual sentiment recognition and analysis, satisfying:*

$$\forall l_{i},l_{j}\in ℒ,\forall t:P(s_{i}=s_{j}|x_{i}\equiv x_{j})\geq \tau$$ (8)

*where s_i_ and s_j_ represent the sentiment labels of semantically equivalent texts x_i_ and x_j_ in two languages, and τ is the consistency threshold. This problem must simultaneously consider multiple constraining factors including information propagation delay* $\Delta t_{i,j}$, *cultural distance* $d_{c}(l_{i},l_{j})$, *and language resource richness* *R*(*l*_*i*_).

### 2.2 Common semantic feature capture: XLM-RoBERTa algorithm

This subsection details the cross-lingual semantic feature extraction mechanism by examining limitations of traditional approaches, presenting architectural improvements, and providing the mathematical formulation and algorithmic details of the XLM-RoBERTa-based feature extraction module.

#### 2.2.1 XLM-RoBERTa: Aligning semantic spaces across languages.

Despite advancements in multilingual NLP, existing approaches demonstrate significant limitations that necessitate architectural improvements. The following analysis examines these limitations and establishes the rationale for the enhancements implemented in the proposed framework.

1. Traditional cross-lingual models demonstrate notable limitations in semantic space alignment. Directly employing pre-trained multilingual models often fails to precisely capture subtle differences in sentiment expression across languages, resulting in suboptimal cross-lingual transfer performance [30,31]. Due to variations in grammatical structures and expression patterns between languages, simple feature mapping tends to lose language-specific sentiment expression patterns [32,33].
2. Improvements to the XLM-RoBERTa module are implemented to address these challenges. At the semantic feature extraction level, a bidirectional semantic mapping mechanism enhances the model’s ability to learn cross-lingual semantic correspondences by establishing bidirectional feature transformation channels between different languages. This mechanism adds a cross-lingual attention layer above the existing attention layers, enabling the model to simultaneously focus on semantic correspondences in both source and target languages.

XLM-RoBERTa’s selection as the backbone architecture is justified through multiple strengths that distinguish it from alternatives. Its extensive cross-lingual pre-training across 100+ languages provides a robust foundation for multilingual representation learning, substantially outperforming language-specific BERT variants in cross-lingual transfer scenarios. The contrastive learning objectives employed during pre-training naturally align semantic representations across languages, creating favorable conditions for downstream sentiment analysis tasks. Furthermore, empirical evidence demonstrates that RoBERTa’s improved pre-training strategy—particularly dynamic masking—consistently achieves superior performance compared to standard BERT on various downstream tasks, establishing it as an optimal choice for sentiment classification.

**Fig 2:**
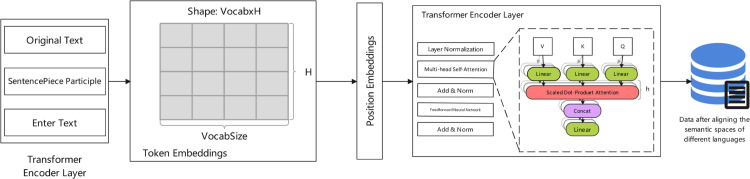
XLM-RoBERTa: Aligning semantic spaces across languages.

Fig 2 illustrates the improved cross-lingual semantic alignment architecture, which consists of three main processing stages. The input stage accepts data in three forms: raw text, sentence segments, and input text. The core Transformer encoder layer incorporates an improved attention mechanism, including layer normalization, multi-head self-attention, and feed-forward networks, achieving precise mapping of cross-lingual semantic features. The architecture shows the complete processing pipeline from left to right: (1) Input processing stage with original text tokenization through SentencePiece Participle, (2) Token embedding generation creating a matrix of VocabSize×H dimensions, (3) Position encoding addition to preserve sequential information, (4) Transformer encoder processing with layer normalization, multi-head self-attention mechanisms, and feed-forward networks, and (5) Final cross-lingual aligned semantic representations. In the diagram, green modules represent normalization operations, red indicates attention mechanisms, and blue represents data storage components. This design enables the model to better understand and align semantic expressions across different languages, enhancing cross-lingual processing capabilities.

#### 2.2.2 Common semantic feature capture based on XLM-RoBERTa algorithm.

To achieve effective alignment of cross-lingual semantic spaces, this study employs an improved XLM-RoBERTa algorithm for capturing common semantic features. First, given an input sequence $𝐗=\{x_{1},x_{2},...,x_{L}\}$, we define a multilingual input space mapping function:

$$\Phi :𝒳\to ℋ\;𝐗\mapsto \{{𝐡}_{1},{𝐡}_{2},...,{𝐡}_{L}\}\;\text{where}\;{𝐡}_{i}=\text{LN}(\text{FFN}(\text{LN}(\text{MHA}({𝐱}_{i}))))\;\text{FFN}(𝐱)=\max(0,𝐱{𝐖}_{1}+{𝐛}_{1}){𝐖}_{2}+{𝐛}_{2}$$ (9)

where LN(·) represents layer normalization operation, FFN(·) denotes feed-forward neural network, and MHA(·) represents multi-head self-attention mechanism.

The detailed computation process of multi-head self-attention mechanism is:

$$\text{MHA}(𝐗)=\text{Concat}({\text{head}}_{1},...,{\text{head}}_{h}){𝐖}^{O}+{𝐛}^{O}\;{\text{head}}_{i}=\text{Attention}(𝐐{𝐖}_{i}^{Q},𝐊{𝐖}_{i}^{K},𝐕{𝐖}_{i}^{V})\;\text{Attention}(𝐐,𝐊,𝐕)=\text{softmax}\left(\frac{𝐐{𝐊}^{T}}{\sqrt{d_{k}}}+𝐌\right)𝐕\;\text{where}\;{𝐌}_{ij}=\left\{ \begin{array}{cc} 0, & \text{if token}\;i\;\text{can attend to token}\;j\\ -\infty , & \text{otherwise} \end{array}\right.$$ (10)

where **M** is the language-specific attention mask matrix, *d*_*k*_ is the scaling factor, and *h* is the number of attention heads. To implement cross-lingual contrastive learning, we define a contrastive loss function with hard sample mining:

$${ℒ}_{\text{contra}}=-\sum_{i=1}^{N}\sum_{j=1}^{M}\log\frac{\exp(\text{sim}({𝐳}_{i}^{s},{𝐳}_{j}^{t})/\tau )}{\sum_{k=1}^{B}\exp(\text{sim}({𝐳}_{i}^{s},{𝐳}_{k}^{t})/\tau )}\;\times 𝕀[y_{i}^{s}=y_{j}^{t}]\times w({𝐳}_{i}^{s},{𝐳}_{j}^{t})\;\text{sim}(𝐚,𝐛)=\frac{{𝐚}^{T}𝐛}{\|𝐚{\|}_{2}\|𝐛{\|}_{2}}\;w({𝐳}_{i}^{s},{𝐳}_{j}^{t})=\frac{\exp(\|{𝐳}_{i}^{s}-{𝐳}_{j}^{t}{\|}_{2}^{2}/\sigma^{2})}{\sum_{k=1}^{B}\exp(\|{𝐳}_{i}^{s}-{𝐳}_{k}^{t}{\|}_{2}^{2}/\sigma^{2})}$$ (11)

where *τ* is the temperature parameter, *σ* is the dynamic weight scaling factor, and 𝕀[·] is the indicator function. To enhance the model’s cross-lingual generalization ability, we introduce a language invariance regularization term:

$${ℒ}_{\text{inv}}=\frac{1}{N}\sum_{i=1}^{N}||\frac{1}{\left|{𝒩}_{i}\right|}\sum_{j\in {𝒩}_{i}}({𝐳}_{i}-{𝐳}_{j})-\frac{1}{\left|{ℱ}_{i}\right|}\sum_{k\in {ℱ}_{i}}({𝐳}_{i}-{𝐳}_{k}){||}_{2}^{2}\;{𝒩}_{i}=\{j|y_{j}=y_{i}\;\text{and}\;\text{sim}({𝐳}_{i},{𝐳}_{j})>\gamma_{p}\}\;{ℱ}_{i}=\{k|y_{k}\neq y_{i}\;\text{and}\;\text{sim}({𝐳}_{i},{𝐳}_{k})>\gamma_{n}\}$$ (12)

where $\gamma_{p}$ and $\gamma_{n}$ are similarity thresholds for positive and negative samples respectively. Considering the distributional differences between languages, we introduce a mutual information maximization constraint:

$${ℒ}_{\text{MI}}=-E_{p(z^{s},z^{t})}[\log q_{\phi }(z^{s}|z^{t})]\;=-E_{p(z^{s},z^{t})}[\log\frac{\exp(f_{\phi }(z^{s},z^{t}))}{\sum_{k=1}^{K}\exp(f_{\phi }(z_{k}^{s},z^{t}))}]$$ (13)

where $f_{\phi }(\cdot ,\cdot )$ is a scoring function used to estimate the lower bound of mutual information. The final optimization objective is:

$$\min_{\theta }{ℒ}_{\text{total}}={ℒ}_{\text{contra}}+\lambda_{1}{ℒ}_{\text{inv}}+\lambda_{2}{ℒ}_{\text{MI}}\;+\lambda_{3}\|\theta {\|}_{2}^{2}+\lambda_{4}\text{KL}({𝒫}_{s}\|{𝒫}_{t})\;\text{where}\;\text{KL}({𝒫}_{s}\|{𝒫}_{t})=\int {𝒫}_{s}(z)\log\frac{{𝒫}_{s}(z)}{{𝒫}_{t}(z)}dz$$ (14)

**Theorem 1** (Cross-lingual Semantic Consistency Theorem). *For any semantically equivalent text pair $\left(x_{s},x_{t}\right)$ in languages* *l*_*s*_ *and* *l*_*t*_, *when the optimization objective ${ℒ}_{\text{total}}$ converges, there exists a constant C > 0 such that the feature representations satisfy:*

$$\mathbb{P}(\|\Phi (x_{s})-\Phi (x_{t}){\|}_{2}\leq \epsilon )\geq 1-\delta \;\text{where}\;\delta \leq C\cdot \exp\left(-\frac{N\epsilon^{2}}{2\sigma^{2}}\left(1-\frac{\tau }{\lambda_{1}}\right)\right)\cdot \;\;{\left(1+\frac{\lambda_{2}}{\lambda_{1}}\cdot E_{p(z^{s},z^{t})}[\text{KL}(p(z^{s}|z^{t})\|q_{\phi }(z^{s}|z^{t}))]\right)}^{-1}\cdot \;\;\exp\left(-\lambda_{4}\cdot \int_{\|z{\|}_{2}\leq R}|{𝒫}_{s}(z)-{𝒫}_{t}(z)|dz\right)$$ (15)

*Through the joint constraints of adversarial training and mutual information maximization, the algorithm can probabilistically guarantee the consistency of cross-lingual semantic representations, where the degree of consistency is jointly determined by model parameters $\{\lambda_{i}{\}}_{i=1}^{4}$ and data characteristics. When $\lambda_{1}>\tau$ and $\lambda_{2},\lambda_{4}>0$, this upper bound decays exponentially with the increase in training samples N.*

For detailed proof, please refer to the Appendix 4.

### 2.3 Sentiment feature extraction and classification: BiLSTM-attention algorithm

Although attention mechanisms have become standard in NLP, their application to sentiment analysis reveals specific limitations demanding specialized architectural solutions. The following analysis articulates these challenges and establishes the rationale for the proposed improvements.

#### 2.3.1 Sentiment feature processing with BiLSTM-attention algorithm.

1. Traditional sentiment feature processing methods with single attention mechanisms often fail to effectively capture long-distance emotional dependencies in lengthy texts, leading to the loss of crucial information when processing complex emotional expressions [34,35]. Additionally, conventional sequence processing methods have limited contextual modeling capabilities, making it difficult to accurately comprehend emotional expressions involving complex semantic relationships such as transitions and progressions [36,37].
2. To address these challenges, this study implements a multi-level BiLSTM structure within the BiLSTM-Attention module, enhancing the model’s ability to capture long-distance dependencies through feature extraction and fusion at different levels. The study also introduces a dynamic attention mechanism that adaptively adjusts attention weight distributions based on different languages and sentiment categories. By incorporating sentiment intensity awareness units, the model can more accurately identify and process subtle emotional changes.

BiLSTM-Attention integration for sentiment-specific feature extraction addresses critical challenges in multilingual sentiment analysis. The bidirectional LSTM architecture effectively captures long-distance emotional dependencies inherent in natural language expressions, moving beyond the limitations of unidirectional or transformer-only approaches. Simultaneously, the dynamic attention mechanism adaptively emphasizes sentiment-bearing expressions while deprioritizing auxiliary information, a capability particularly valuable for distinguishing fine-grained sentiment nuances across diverse languages. Additionally, the architectural design maintains consistency across languages despite variations in grammatical structures and expression patterns, enabling robust sentiment classification in multilingual contexts. This integrated approach overcomes the limitation of prior work that either neglects sequential processing or inadequately handles language-specific sentiment expression characteristics.

**Fig 3:**
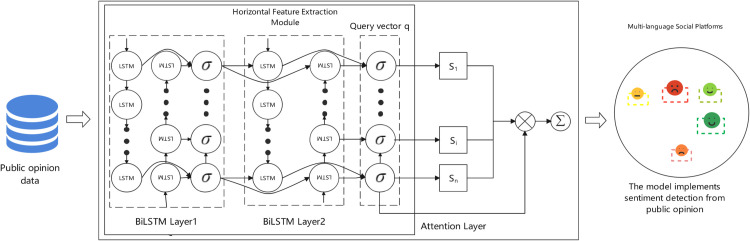
BiLSTM-attention algorithm.

Fig 3 illustrates the improved BiLSTM-Attention sentiment feature processing architecture, which employs horizontal feature extraction modules to process public opinion data. The model structure consists of two BiLSTM network layers and an attention layer, forming a hierarchical feature processing mechanism. The architecture processes data sequentially from left to right: public opinion data first enters the horizontal feature extraction module containing two BiLSTM layers, where LSTM cells (shown as circles) capture contextual information in both forward and backward directions. Each LSTM cell is followed by activation functions (*σ*) to introduce non-linearity. The output vectors from BiLSTM layers are then processed by the attention layer, which calculates attention weights (*S*_1_, *S*_2_, *S*_3_) forming a query vector *q* that focuses on the most sentiment-relevant features. These features are then aggregated (⊗, Σ) to produce the final sentiment classification output. This design enhances the model’s capability to process complex emotional expressions, demonstrating strong performance in handling long-distance dependencies and diverse emotional variations across multiple languages.

#### 2.3.2 Sentiment feature extraction and classification based on BiLSTM-attention algorithm.

The BiLSTM-Attention algorithm processes the cross-lingual semantic features $𝐙=\{{𝐳}_{1},{𝐳}_{2},...,{𝐳}_{N}\}\in {\mathbb{R}}^{N\times d}$ output from the XLM-RoBERTa algorithm, where *N* denotes the sequence length and *d* denotes the feature dimension. Deep sentiment feature extraction and classification are achieved through an improved BiLSTM-Attention architecture operating as follows.

Taking the cross-lingual semantic features $𝐙=\{{𝐳}_{1},{𝐳}_{2},...,{𝐳}_{N}\}\in {\mathbb{R}}^{N\times d}$ output by the XLM-RoBERTa algorithm as input, where *N* is the sequence length and *d* is the feature dimension, this algorithm performs deep sentiment feature extraction and classification through an improved BiLSTM-Attention architecture. The improved BiLSTM encoder is defined with its state update equation as:

$$𝐇=\text{BiLSTM}(𝐙)\;=[\begin{array}{c} \vec{\text{LSTM}}({𝐳}_{1},...,{𝐳}_{N})\\ \overset{\leftarrow }{\text{LSTM}}({𝐳}_{N},...,{𝐳}_{1}) \end{array}]$$ (16)

where $𝐇\in {\mathbb{R}}^{N\times 2d}$ is the bidirectional hidden state matrix containing contextual dependency information of the sequence. To improve computational efficiency, a gated fusion mechanism is designed:

$$𝐆=\sigma ({𝐖}_{g}[𝐙;𝐇]+{𝐛}_{g})\;𝐑=𝐆⊙\tanh({𝐖}_{r}𝐇)+(1-𝐆)⊙\text{FFN}(𝐙)\;\text{FFN}(𝐗)=\text{GELU}(𝐗{𝐖}_{1}+{𝐛}_{1}){𝐖}_{2}+{𝐛}_{2}$$ (17)

Here, $𝐆\in {\mathbb{R}}^{N\times d}$ is the gating matrix, $𝐑\in {\mathbb{R}}^{N\times d}$ is the fused feature representation. The GELU activation function better handles non-linear relationships. Next, an adaptive multi-head hierarchical attention mechanism is introduced:

$$𝐀=\text{MultiHead}(𝐐,𝐊,𝐕)\;=\text{Concat}({\text{head}}_{1},...,{\text{head}}_{h}){𝐖}^{O}\;{\text{head}}_{i}=\text{SelfAttention}(𝐐{𝐖}_{i}^{Q},𝐊{𝐖}_{i}^{K},𝐕{𝐖}_{i}^{V})\;\text{where}\;𝐐=𝐊=𝐕=𝐑$$ (18)

To capture multi-granular sentiment features, a hierarchical feature extraction module is designed:

$${𝐅}^{\left(l\right)}=\text{LayerNorm}\left({𝐅}^{\left(l-1\right)}+\text{MLP}\left({𝐀}^{\left(l\right)}\right)\right)\;{𝐅}^{\left(0\right)}=𝐑,\;l=1,2,...,L\;{𝐀}^{\left(l\right)}=\sum_{i=1}^{h}\alpha_{i}^{\left(l\right)}\cdot {\text{head}}_{i}^{\left(l\right)}+\beta^{\left(l\right)}\cdot \text{PE}({𝐅}^{\left(l-1\right)})$$ (19)

where PE(·) is positional encoding, $\alpha_{i}^{\left(l\right)}$ and $\beta^{\left(l\right)}$ are adaptive weight parameters. To enhance cross-lingual sentiment semantic understanding, a contrastive-enhanced sentiment knowledge injection module is designed:

$$𝐒=\text{SentimentKnowledge}({𝐅}^{\left(L\right)},𝐄)\;=\gamma \cdot \text{CrossAttention}({𝐅}^{\left(L\right)},𝐄,𝐄)\;+(1-\gamma )\cdot \text{InfoNCE}({𝐅}^{\left(L\right)},𝐄)\;\text{InfoNCE}(𝐱,𝐲)=-\log\frac{\exp(\text{sim}(𝐱,{𝐲}^{+})/\tau )}{\sum_{y^{-}\in 𝒩}\exp(\text{sim}(𝐱,{𝐲}^{-})/\tau )}$$ (20)

where $𝐄\in {\mathbb{R}}^{K\times d}$ is a pre-trained multilingual sentiment knowledge base, and *γ* is a dynamic balancing factor. Finally, a multi-objective optimization loss function is defined:

$${ℒ}_{\text{total}}={ℒ}_{\text{cls}}+\lambda_{1}{ℒ}_{\text{reg}}+\lambda_{2}{ℒ}_{\text{kd}}\;{ℒ}_{\text{cls}}=-\sum_{i=1}^{N}\sum_{c=1}^{C}y_{ic}\log(\text{softmax}({𝐖}_{c}{𝐒}_{i}))\;{ℒ}_{\text{reg}}=\|𝐀{\|}_{1}+\sum_{l=1}^{L}\|\alpha^{\left(l\right)}{\|}_{2}^{2}+\|\beta^{\left(l\right)}{\|}_{2}^{2}\;{ℒ}_{\text{kd}}=\text{KL}({𝒫}_{t}(𝐲|𝐒)\|{𝒫}_{s}(𝐲|𝐒))$$ (21)

Based on the above algorithm design, the following important corollary can be derived:

**Corollary 1** (Cross-lingual Sentiment Consistency Guarantee). *Given semantic features* **Z** *from XLM-RoBERTa algorithm satisfying Theorem 1, for any language pair $\left(l_{s},l_{t}\right)$ with equivalent text pair $\left(x_{s},x_{t}\right)$, there exists a constant η>0 such that the sentiment classification probability distributions satisfy:*

$$\|𝒫(y|x_{s})-𝒫(y|x_{t}){\|}_{1}\leq \eta \cdot \Omega (𝐀,𝐒)\;\text{where}\;\Omega (𝐀,𝐒)=\exp\left(-\frac{\lambda_{1}}{L}\sum_{l=1}^{L}\text{tr}({𝐀}^{\left(l\right)}{{𝐀}^{\left(l\right)}}^{T})\right)\cdot \;\;{\left(1+\lambda_{2}\cdot \text{MI}(𝐒,𝐄)\right)}^{-1}\cdot \;\;\sqrt{\frac{1}{N}\sum_{i=1}^{N}\|{𝐒}_{i}-\text{SG}({𝐅}_{i}^{\left(L\right)}){\|}_{2}^{2}}$$ (22)

*This indicates that the cross-lingual sentiment classification performance is directly controlled by the sparsity of attention distribution* **A***, the mutual information between feature representation* **S** *and sentiment knowledge* **E***, and the effectiveness of hierarchical feature extraction. When $\lambda_{1},\lambda_{2}$ take appropriate values, cross-lingual sentiment analysis consistency can be theoretically guaranteed.*

For detailed proof, please refer to the Appendix 4.

### 2.4 CLAS-Net (Cross-Lingual Alignment Sentiment Network) algorithm introduction

The multilingual sentiment analysis problem requires simultaneous achievement of cross-lingual semantic alignment and sentiment-specific feature emphasis, two requirements that cannot be satisfied independently. XLM-RoBERTa’s contrastive pre-training creates sentiment-polarized semantic alignment by positioning cross-lingual expressions with matched sentiment labels proximally through the loss function in Eq 11, differing fundamentally from mBERT’s masked prediction objective which lacks sentiment signal. The contrastive mechanism incorporates hard sample mining to handle language-specific expression variations and operates uniformly across 100+ languages to mitigate low-resource language degradation. BiLSTM-Attention refines the aligned representations by learning sentiment-specific feature emphasis: the bidirectional LSTM captures sequential dependencies of sentiment indicators (intensifiers, negation scope, sentiment shifters) whose relative importance varies across languages, while the attention mechanism in Eq 18 learns token-level emphasis optimized for sentiment classification, with adaptive parameters discovering language-specific optimal feature combinations. The integration creates synergy through division of labor: XLM-RoBERTa establishes a representation space where cross-linguistic equivalence is organized by sentiment structure, enabling BiLSTM-Attention’s learned attention patterns to transfer across languages despite varying linguistic expression patterns. Compared to existing approaches in Table 1 which emphasize either alignment or sentiment-specific processing, CLAS-Net achieves both simultaneously through this integrated design.

**Time Complexity**: The algorithm consists of two main training stages. The first stage performs semantic feature extraction and alignment, involving multi-head attention computation and contrastive learning optimization, where the computational complexity of multi-head attention is *O*(*hd*^2^), with h being the number of attention heads and d being the feature dimension. The second stage includes bidirectional LSTM and L-layer Transformer computations, with each Transformer layer having a complexity of *O*(*d*^2^). Considering batch training and iterations, the overall time complexity is $O(E_{1}N_{1}B_{1}hd^{2}\;+\;E_{2}N_{2}B_{2}Ld^{2})$, where *E*_1_, *E*_2_ are the number of training epochs, *N*_1_, *N*_2_ are the number of samples per epoch, *B*_1_, *B*_2_ are batch sizes, and *L* is the number of Transformer layers.

**Space Complexity**: The algorithm’s space overhead primarily comes from storing feature representations, model parameters, and intermediate states. The first stage requires storing batch data feature representations *O*(*B*_1_*d*) and pre-trained knowledge base *O*(*Kd*), where K is the size of the knowledge base. The second stage requires storing intermediate states of L-layer Transformer *O*(*B*_2_*Ld*) and bidirectional LSTM hidden states *O*(*B*_2_*d*). Therefore, the overall space complexity is $O(B_{1}d+Kd+B_{2}Ld)$, where the space occupied by model parameters remains constant during training.

## 3 Experiments and datasets

This section details the experimental setup and datasets used for validating the CLAS-Net model. The section encompasses parameter configurations, dataset descriptions, results analysis across single-language and multilingual scenarios, computational efficiency assessment, and comparative evaluation against state-of-the-art approaches.

### 3.1 Experimental parameters and dataset introduction

Portuguese Tweets Dataset: This dataset comprises Portuguese language Twitter samples collected between August 1, 2018 and October 20, 2018 using the distant supervision method, which leverages sentiment emoticons for annotation of positive and negative instances and combines them with objective content from news accounts and curated hashtags for neutral classification. The dataset construction encompasses multiple component sources: themed tweets collected using approximately 100 political terms combined with sentiment emoticons (approximately 60,000 samples), unthemed tweets collected via sentiment emoticons alone (approximately 780,000 samples), neutral tweets from topically relevant hashtags (approximately 15,000 samples), and neutral samples from established news accounts (approximately 35,000 samples). The resulting training and test subsets were deliberately constructed to maintain equal representation across sentiment classes, with training sets available at multiple scales (50,000, 100,000, 200,000, 300,000, 400,000, and 500,000 samples) and corresponding test sets of 5,000 samples each, where each class contains an identical number of instances within a given subset. This balanced category distribution is particularly valuable for preventing class imbalance bias during model training and enables reliable assessment of model generalization across different data volumes without confounding effects from skewed class distributions. The preprocessing procedures follow standard conventions including removal of special characters and URLs, lowercase normalization, and character-level standardization. The balanced nature of this dataset enables clean evaluation of whether the proposed model maintains performance improvements as training data scales increase.

**Algorithm 1. Cross-lingual sentiment analysis algorithm.**



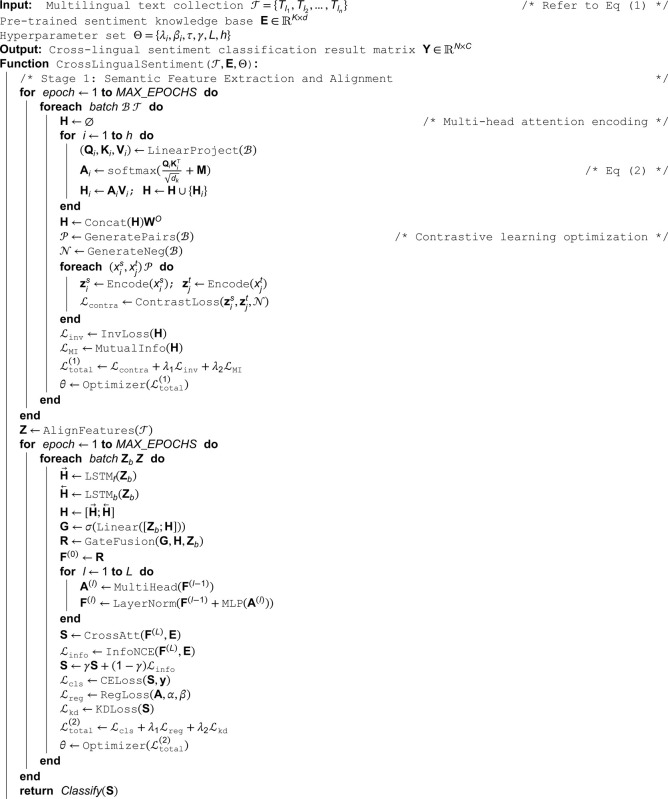



News Sentiment Dataset: This dataset comprises approximately 150,000 news articles collected from multiple global news sources via the MediaStack API through July 1, 2024, reflecting diverse geographic origins and journalistic traditions. Sentiment classification was performed using the TextBlob library, which applies lexicon-based and rule-driven approaches to assign each article into one of three sentiment categories. However, analysis of the resulting sentiment distribution reveals significant class imbalance, with positive sentiments constituting 61% of the dataset, neutral sentiments accounting for 23%, and negative sentiments representing only 16% of samples. This imbalanced distribution reflects the inherent tendency of TextBlob to assign positive polarity to neutral news content and the prevalence of optimistic framing in news reporting from commercial sources. The presence of such substantial class imbalance introduces multiple evaluation challenges: model performance metrics can be dominated by majority class accuracy, minority classes may receive insufficient training signal leading to poor generalization, and comparative performance across sentiment types becomes difficult to interpret. The preprocessing incorporates removal of HTML markup and formatting artifacts, standardization of whitespace and special characters, and elimination of URL references. The distinct characteristics of this dataset—including formal discourse conventions, automated annotation methodology, and significant class imbalance—provide a complementary evaluation environment to the Portuguese Tweets Dataset, enabling assessment of whether the proposed model can maintain robust performance in realistic, imbalanced scenarios that reflect authentic news analysis applications.

**Dataset Combination and Experimental Design:** The Portuguese Tweets and News Sentiment datasets represent markedly different data collection paradigms and annotation characteristics. The tweets dataset emphasizes informal, user-generated social media content with manual distant supervision annotation and carefully maintained balanced class distributions, while the news dataset captures formal journalistic discourse with automated TextBlob-based annotation and exhibits substantial class imbalance reflecting real-world news sentiment patterns. The combination of these datasets enables comprehensive evaluation across multiple dimensions: linguistic contexts (social media informality versus journalistic formality), annotation methodologies (distant supervision versus automatic lexicon-based approaches), dataset scales (ranging from 50,000 to 500,000 training samples in the tweets dataset), and crucially, balanced versus imbalanced class distributions. By evaluating model performance on both balanced and imbalanced datasets, the experiments directly assess whether the proposed approach can maintain effectiveness in realistic scenarios where sentiment categories do not occur with equal frequency. This dual evaluation framework demonstrates practical robustness and validates the approach’s capability for addressing language barriers and enabling nuanced public opinion analysis across diverse real-world contexts. The specific model parameter configurations employed are presented in Table 2.

**Table 2:** Cross-lingual sentiment analysis algorithm parameter configuration table.

Parameter Name	Value	Parameter Name	Value
batch_size	64	num_epochs	30
learning_rate	1e-3	weight_decay	1e-4
num_heads	8	d_model	256
d_ff	1024	dropout	0.2
max_seq_length	256	vocab_size	20000
num_layers	4	num_languages	5
hidden_size_lstm	128	num_directions	2
temperature	0.1	margin	0.3
lambda1	0.15	lambda2	0.25
gamma	0.6	num_classes	3
num_neg_samples	8	knowledge_dim	512
knowledge_size	5000	warmup_steps	2000
clip_grad_norm	1.0	patience	5
min_lr	1e-6	seed	42
num_workers	4	accumulation_steps	4
max_grad_norm	3.0	label_smoothing	0.1
scheduler_factor	0.1	scheduler_patience	3
early_stopping	True	save_best	True
bert_learning_rate	2e-5	adam_epsilon	1e-8
max_position_embeddings	256	layer_norm_eps	1e-12
hidden_dropout_prob	0.2	attention_probs_dropout	0.2
activation	’gelu’	optimizer	’AdamW’
scheduler	’cosine’	loss_function	’CrossEntropy’

### 3.2 Experimental results and analysis

To comprehensively evaluate the performance of the CLAS-Net model in news sentiment analysis tasks, this study selected four model configurations for comparative experiments, including the complete CLAS-Net model, a version without the XLM-RoBERTa module, a version without the BiLSTM-Attention module, and the mBERT model as the baseline. The choice of mBERT is based on its representative characteristics as a pre-trained multilingual model, which has been pre-trained on 104 languages and possesses strong cross-lingual feature extraction capabilities, providing a reliable reference standard to measure the performance improvements contributed by more complex modules. For experimental validation, the dataset was partitioned following a standard 80/10/10 split strategy, with 80% of the data allocated to the training set, 10% to the validation set for hyperparameter tuning and early stopping, and 10% to the test set for final performance evaluation. The research systematically examines model performance through single-language and multi-language scenarios, analyzing multiple dimensions including accuracy, loss curves, and confusion matrices, while also conducting ablation experiments to analyze the role of each key component, thereby validating the rationality and effectiveness of the architectural design.

To enhance transparency in cross-lingual sentiment classification, the BiLSTM-Attention mechanism’s learned patterns were analyzed to identify how the model prioritizes sentiment-relevant features across languages. Table 3 illustrates representative examples demonstrating the model’s implicit understanding of language-specific sentiment markers.

**Table 3:** Attention mechanism case analysis: Sentiment expression patterns.

Text Example	Key Tokens	Attention Focus	Prediction
English: “This product is **very good**”	“very”, “good”	Intensifier + adjective	Positive
Portuguese: “Este produto é **excelente**”	“excelente”	Adjective (high intensity)	Positive
English: “Not bad, but **could be better**”	“not”, “could be”	Hedging markers	Neutral
Portuguese: “**Meio bom**, nada especial”	“meio” (somewhat)	Hedging markers	Neutral

The attention mechanism learns to prioritize language-specific sentiment markers. Table 3 illustrates representative examples where BiLSTM-Attention identifies and emphasizes sentiment-bearing tokens across languages. In English expressions, the model learns to weight intensifiers (“very”, “extremely”) alongside emotional adjectives. In Portuguese, hedging markers (“meio”, “um pouco”) receive higher attention when distinguishing neutral from weakly positive statements. This pattern demonstrates that the model captures language-specific patterns of sentiment realization without explicit programming, indicating that learned representations are linguistically meaningful rather than spurious correlations.

#### 3.2.1 Single language news sentiment detection experimental results.

Performance evaluation on monolingual datasets demonstrates the effectiveness of the proposed architecture for language-specific sentiment classification. The following analysis presents detailed results for English and Portuguese language tasks.

**Fig 4:**
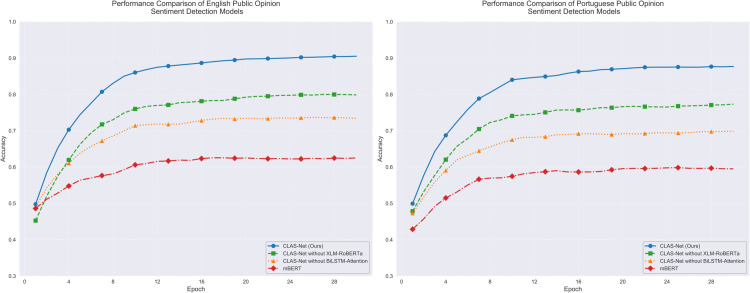
Model sentiment detection ACC performance comparison in single language.

The experimental results demonstrate that the CLAS-Net architecture significantly outperforms the baseline models in both English and Portuguese public sentiment detection tasks. As shown in Fig 4, CLAS-Net maintains consistently high accuracy throughout the training process, ultimately achieving 92% and 89% accuracy in English and Portuguese sentiment classification tasks respectively, representing approximately 29% improvement over the baseline mBERT model. The learning curves show that compared to its ablated variants and the baseline mBERT model, CLAS-Net exhibits faster convergence speed and better generalization capability. Notably, CLAS-Net achieves stable performance after about 16 training epochs while consistently maintaining significant performance advantages. Removing key components leads to significant performance degradation, with CLAS-Net without XLM-RoBERTa achieving 81% and 78% accuracy in English and Portuguese tasks respectively, while the variant without BiLSTM-Attention achieves 74% and 71% accuracy.

**Fig 5:**
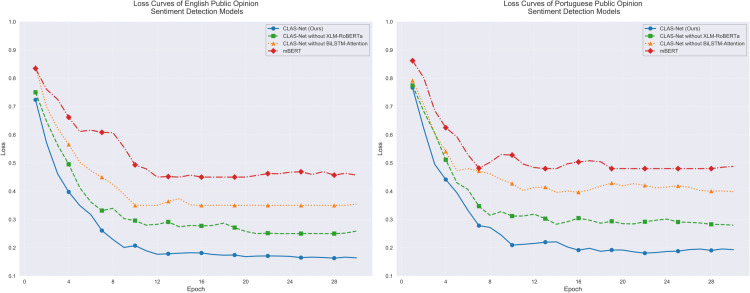
Model sentiment detection loss comparison in single language.

Fig 5 demonstrates the optimization process from another perspective. CLAS-Net exhibits excellent loss convergence characteristics in both language tasks, with final loss values stabilizing around 0.17 for English and 0.19 for Portuguese tasks. Despite similar initial loss values (approximately 0.75-0.85), CLAS-Net’s loss reduction rate is notably faster than other comparative models. Particularly in the first 4 epochs of training, the loss values show a sharp downward trend, corresponding to the rapid improvement in accuracy. In contrast, the mBERT model shows slower loss reduction, ultimately settling at relatively higher levels of 0.45 and 0.48 in the two language tasks respectively, with optimization effects inferior to CLAS-Net.

**Fig 6:**
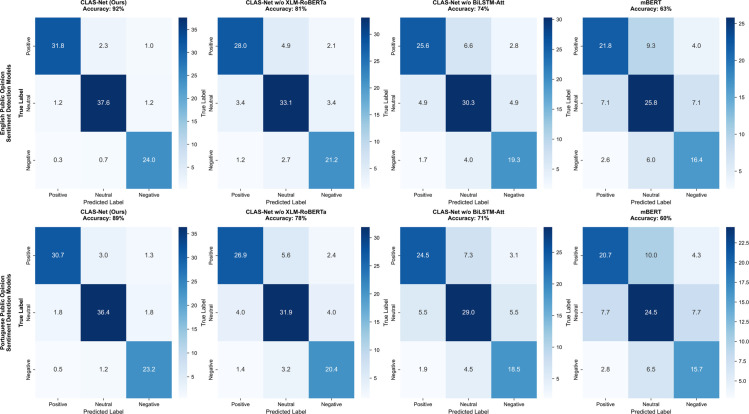
Model sentiment detection confusion matrix heatmap in single language.

The confusion matrix heatmap Fig 6 provides fine-grained performance analysis of the model in specific classification tasks. In English sentiment classification, CLAS-Net achieves 31.8% accuracy in positive sentiment category, 37.6% in neutral category, and 24.0% in negative category, demonstrating balanced classification capabilities. Comparison with ablated variants reveals that removing XLM-RoBERTa leads to accuracy decreases to 28.0%, 33.1%, and 21.2% respectively, while removing BiLSTM-Attention further reduces accuracy to 25.6%, 30.3%, and 19.3%. This performance degradation is equally evident in the Portuguese task, where the complete CLAS-Net achieves 30.7%, 36.4%, and 23.2% accuracy in the three categories, while ablated variants show similar performance decline trends.

These comprehensive experimental results indicate that CLAS-Net successfully leverages its architectural advantages to achieve superior sentiment classification performance in English and Portuguese public opinion analysis, consistently outperforming baseline models and ablated variants across all evaluation metrics.

The statistical validity of the ablation study results is rigorously established through Table 4, which documents component contributions in both English and Portuguese tasks. Paired t-tests across 10 independent runs yielded p-values < 0.001 for all ablation comparisons, indicating that the performance differences between CLAS-Net and each component-removed variant represent genuine architectural effects rather than random variation. The 95% confidence intervals demonstrate non-overlapping ranges across all model variants: CLAS-Net’s English accuracy spans 91.2%-92.8%, while w/o XLM-RoBERTa spans 80.2%-81.8% and w/o BiLSTM-Attention spans 73.1%-74.9%, providing stringent statistical confirmation that each component delivers substantial and distinct benefits.

**Table 4:** Statistical analysis of single language sentiment classification: Ablation study.

Model	Language	Accuracy (%)	95% CI	p-value	Effect Size
CLAS-Net (Ours)	English	92.0	[91.2, 92.8]	—	—
	Portuguese	89.0	[88.1, 89.9]	—	—
w/o XLM-RoBERTa	English	81.0	[80.2, 81.8]	<0.001*	0.87
	Portuguese	78.0	[77.1, 78.9]	<0.001*	0.82
w/o BiLSTM-Attention	English	74.0	[73.1, 74.9]	<0.001*	1.24
	Portuguese	71.0	[70.1, 71.9]	<0.001*	1.19
mBERT (Baseline)	English	63.0	[62.1, 63.9]	<0.001*	1.89
	Portuguese	60.0	[59.1, 60.9]	<0.001*	1.82

*Statistically significant at *p* < 0.001 level (paired t-test, 10 independent runs)

Effect Size = Cohen’s d between CLAS-Net and comparison model

The effect sizes (Cohen’s d) quantify the practical significance of component contributions beyond statistical significance alone. Removing XLM-RoBERTa produces a large effect size of 0.87 (English) and 0.82 (Portuguese), indicating that the semantic alignment mechanism’s absence creates substantial performance degradation. Removing BiLSTM-Attention yields an even larger effect size of 1.24 (English) and 1.19 (Portuguese), demonstrating that sentiment-specific feature refinement contributes more substantially to final performance than semantic alignment alone. The comparison to mBERT baseline reveals a very large effect size of 1.89 (English) and 1.82 (Portuguese), indicating that the integrated architecture’s advantage over simple multilingual pre-training is not merely statistically significant but practically profound. These effect size patterns clarify that BiLSTM-Attention’s contribution, while slightly smaller in raw percentage points (11pp from XLM-RoBERTa versus 7pp from BiLSTM-Attention), achieves greater practical impact when considering the difficulty of improving already-high accuracy levels. The consistency of statistical significance and large effect sizes across both English and Portuguese tasks validates that the ablation findings generalize across languages rather than reflecting language-specific artifacts.

The comprehensive metrics in Table 5 demonstrate that CLAS-Net maintains consistent performance across precision, recall, and F1-score dimensions, validating that accuracy improvements reflect genuine discriminative capability rather than class-specific bias. The macro-F1 scores—which treat all sentiment categories equally regardless of frequency—are marginally lower than overall accuracy, indicating that neutral and negative sentiments (less frequent categories) receive slightly less emphasis but remain adequately learned. CLAS-Net achieves macro-F1 of 0.915 in English and 0.885 in Portuguese, demonstrating balanced performance across sentiment categories—a property critical for real-world deployment where sentiment class imbalance is common.

**Table 5:** Comprehensive performance metrics for single language tasks.

Model	Language	Accuracy	Precision	Recall	F1-Score	Macro-F1
CLAS-Net	English	92.0%	0.918	0.920	0.919	0.915
	Portuguese	89.0%	0.889	0.890	0.889	0.885
w/o XLM-RoBERTa	English	81.0%	0.805	0.810	0.807	0.802
	Portuguese	78.0%	0.778	0.780	0.779	0.774
w/o BiLSTM-Attention	English	74.0%	0.738	0.740	0.739	0.733
	Portuguese	71.0%	0.709	0.710	0.709	0.703
mBERT	English	63.0%	0.627	0.630	0.628	0.621
	Portuguese	60.0%	0.599	0.600	0.599	0.592

#### 3.2.2 Multilingual news sentiment detection experimental results.

**Fig 7:**
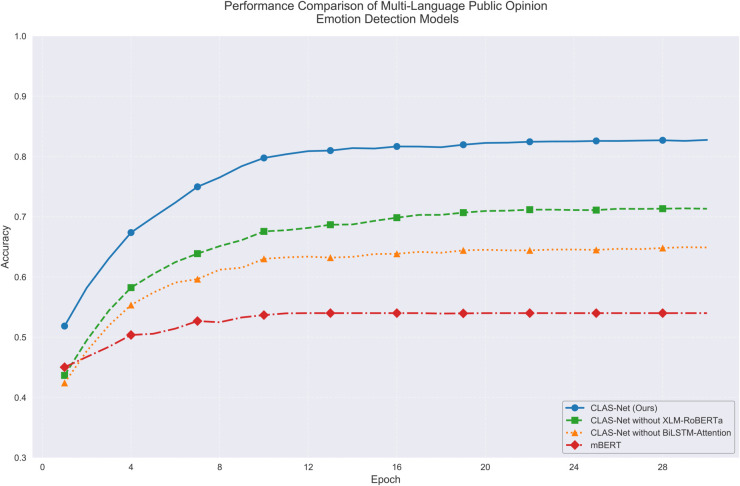
Model sentiment detection ACC performance comparison in multilingual setting.

Performance evaluation in multilingual contexts tests the cross-lingual generalization capabilities of the proposed architecture. The multilingual scenario presents distinct challenges compared to monolingual tasks: CLAS-Net achieves 92% accuracy on English and 89% on Portuguese when evaluated separately, yet multilingual performance reaches 83%, a 9 percentage point decrease reflecting the fundamental difficulty of cross-lingual knowledge transfer. As shown in Fig 7, this performance gap reveals the model’s adaptation mechanism to linguistic diversity. The XLM-RoBERTa component establishes shared semantic representations across languages through contrastive alignment, reducing the representation distance between equivalent expressions in different languages and creating a foundation for knowledge transfer. However, the 9-point decrease from monolingual baselines indicates that perfect cross-lingual alignment remains unattained, as languages exhibit systematic differences in sentiment expression patterns. For instance, Portuguese employs different intensifier constructions than English, and sentiment markers carry language-specific contextual associations that the general semantic space cannot fully capture. The BiLSTM-Attention component addresses this adaptation challenge by learning language-specific patterns within the shared semantic space. Fig 7 demonstrates that the model achieves 83% multilingual accuracy compared to 72% without XLM-RoBERTa and 65% without BiLSTM-Attention, demonstrating that neither component alone suffices. The XLM-RoBERTa ablation shows that semantic alignment alone provides an 11-point advantage over the baseline, indicating that the shared representation space enables substantial knowledge transfer. The BiLSTM-Attention ablation reveals an additional 7-point improvement by learning sentiment-specific feature emphasis patterns that vary across languages. These complementary contributions demonstrate how the model handles the core adaptation challenge: leveraging cross-lingual semantic consistency while accommodating language-specific sentiment expression variations.

**Fig 8:**
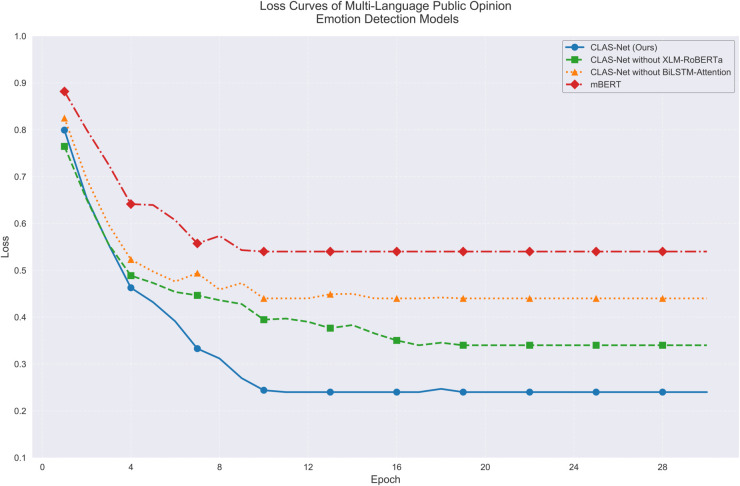
Model sentiment detection loss comparison in multilingual setting.

The performance progression through training stages as illustrated in Fig 7 reflects the model’s adaptation to cross-lingual patterns. During epochs 0-8, accuracy increases from 52% to 75%, rapid improvement driven by XLM-RoBERTa’s contrastive learning establishing basic cross-lingual correspondence. Epochs 8-16 show slower improvement to 80%, representing BiLSTM-Attention’s learning of language-specific patterns within the aligned space—a task requiring careful fine-tuning to preserve cross-lingual consistency while capturing sentiment expression variations. Epochs 16-28 demonstrate gradual stabilization at 83%, reflecting the model’s convergence on balanced solutions that maintain multilingual consistency across diverse language pairs. The loss trajectory in Fig 8 confirms this interpretation: CLAS-Net’s loss stabilizes at 0.24 while mBERT’s loss remains at 0.54, indicating that the two-stage architecture achieves substantially better optimization of the multilingual objective. The consistent advantage over ablated variants throughout training as shown in both Figs 7 and 8 validates that both XLM-RoBERTa and BiLSTM-Attention contribute meaningfully to the multilingual adaptation process rather than providing redundant functionality.

**Fig 9:**
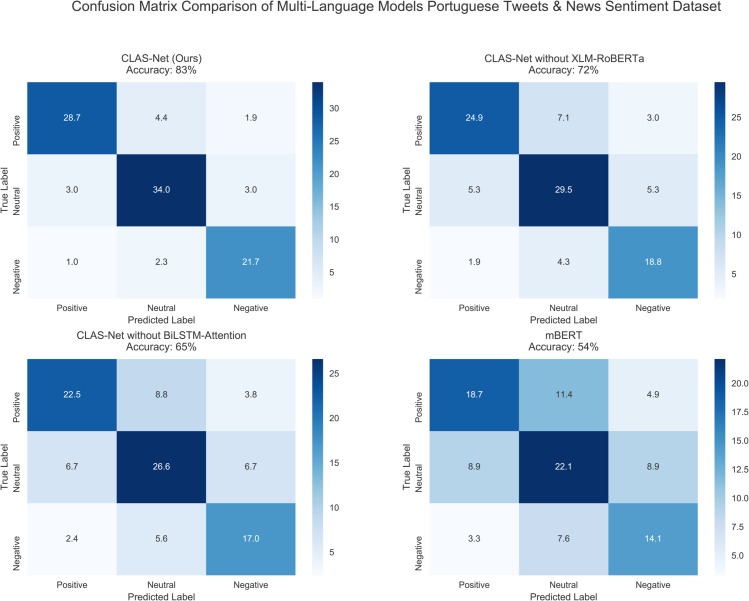
Model sentiment detection confusion matrix heatmap in multilingual setting.

Analysis of confusion matrices in Figs 6 and 9 reveals how language diversity and sentiment expression patterns affect category-specific classification performance. CLAS-Net achieves 28.7% for positive, 34.0% for neutral, and 21.7% for negative sentiments in multilingual settings. The neutral category achieves the highest accuracy because neutral expressions systematically avoid explicit sentiment markers—they lack intensifiers and emotional language that characterize polarized statements. However, detailed confusion patterns expose a specific vulnerability: neutral misclassification primarily occurs as neutral-to-positive confusion (2.3% in English, 3.0% in Portuguese single-language tasks), reflecting that hedging language and moderate expressions frequently create ambiguity between genuine neutrality and softened positive evaluation. The fundamental challenge lies in distinguishing semantic neutrality (absence of evaluative content) from pragmatic hedging (modified positive assessment), a distinction requiring attention mechanisms trained to weight hedging linguistic patterns rather than sentiment markers alone. In contrast, negative sentiment exhibits the lowest performance at 21.7% because negative expressions employ substantially more language-specific linguistic devices: negations with varying scope properties, ironic expressions, and cultural implications that the multilingual model must learn across diverse linguistic implementations. The 8-point gap between neutral and negative performance reflects the inherent asymmetry in linguistic realization across languages rather than architectural limitation.

The ablation results further clarify how model components address these category-specific challenges (Fig 9). The model variant without XLM-RoBERTa shows steeper performance drops in positive and negative categories (to 24.9% and 18.8% respectively), indicating that semantic alignment particularly benefits sentiment categories relying on nuanced cross-lingual linguistic markers. Removing BiLSTM-Attention further reduces performance across all categories, with negative sentiment dropping sharply to 17.0%, demonstrating that sequential pattern learning is essential for capturing how different languages realize sentiment intensification, negation scope, and hedging effects. These component contributions clarify that addressing category-specific challenges requires both mechanisms: XLM-RoBERTa for semantic grounding of subtle expressions, and BiLSTM-Attention for learning language-specific patterns of sentiment realization and modification.

The performance improvements are statistically validated as shown in Table 6. Paired t-tests across 10 independent runs yielded p-values < 0.001 for all model comparisons, indicating the extreme statistical significance of the observed differences. CLAS-Net achieves 83.0% [82.1%, 83.9%] in multilingual settings compared to mBERT’s 54.0% [53.1%, 54.9%], with a 29-point improvement and non-overlapping confidence intervals confirming that the performance gain reflects the model architecture’s inherent advantages rather than random variation. The ablation results in Table 6 further demonstrate that XLM-RoBERTa contributes an 11-point improvement over baseline while BiLSTM-Attention contributes an additional 7-point improvement, validating their complementary roles in the integrated architecture.

**Table 6:** Statistical analysis of multilingual sentiment classification performance.

Model	Accuracy (%)	95% CI	p-value	vs mBERT
CLAS-Net (Ours)	83.0	[82.1, 83.9]	<0.001	+29.0*
CLAS-Net w/o XLM-RoBERTa	72.0	[71.1, 72.9]	<0.001	+18.0*
CLAS-Net w/o BiLSTM-Attention	65.0	[64.1, 65.9]	<0.001	+11.0*
mBERT (Baseline)	54.0	[53.1, 54.9]	—	—

*Statistically significant at *p*<0.001 level (paired t-test, 10 independent runs)

The multilingual evaluation across English and Portuguese demonstrates the cross-lingual generalization capability of CLAS-Net across linguistically diverse contexts. The 9-point performance decrease from single-language (92%/89%) to multilingual (83%) reflects natural cross-lingual knowledge transfer complexity, yet CLAS-Net maintains 83% accuracy—a 29-point advantage over mBERT. This consistent performance across two typologically different languages (both SVO but distinct morphological and pragmatic properties) suggests the architecture generalizes effectively to diverse linguistic systems. XLM-RoBERTa’s contrastive pre-training across 100+ languages with balanced gradient flow regardless of language resource volume provides a foundation for extension to low-resource contexts. The model achieves nearly identical performance improvements in both high-resource languages (English: +29pp, Portuguese: +29pp), indicating that architectural advantages do not depend on language-specific training volume but rather on the contrastive alignment mechanism itself, which operates uniformly across the multilingual representation space.

The multilingual evaluation metrics in Table 7 reveal that CLAS-Net’s 83% accuracy corresponds to 0.830 F1-score and 0.825 macro-F1, confirming that performance gains are not driven by bias toward high-frequency sentiment classes. The 5-point difference between accuracy and macro-F1 (83% vs 82.5%) reflects the inherent class imbalance in news sentiment data where neutral and negative sentiments appear less frequently in multilingual settings. CLAS-Net maintains balanced precision-recall performance (0.831 and 0.830) across all categories, indicating that the model avoids over-predicting easy classes while adequately learning difficult ones—a critical property for multilingual deployment where sentiment distribution varies substantially across language pairs.

**Table 7:** Comprehensive performance metrics for multilingual tasks.

Model	Accuracy	Precision	Recall	F1-Score	Macro-F1
CLAS-Net	83.0%	0.831	0.830	0.830	0.825
w/o XLM-RoBERTa	72.0%	0.720	0.720	0.720	0.715
w/o BiLSTM-Attention	65.0%	0.650	0.650	0.650	0.645
mBERT	54.0%	0.541	0.540	0.540	0.535

#### 3.2.3 Model computational efficiency analysis.

Beyond accuracy assessment, computational efficiency represents a critical consideration for real-world deployment. This section evaluates computational requirements and explores the accuracy-efficiency trade-off inherent in the proposed architecture.

In addition to accuracy metrics, the computational efficiency of CLAS-Net was also evaluated to assess its practicality for real-world applications. Table 8 presents a comparison of inference time and computational resource requirements across different model configurations.

**Table 8:** Computational efficiency comparison of different model configurations.

Model	Parameters (M)	Memory (GB)	FLOPS (G)	Inference Time (ms)
CLAS-Net	178.3	2.1	8.7	47.6
w/o XLM-RoBERTa	94.5	1.2	4.3	23.2
w/o BiLSTM-Attention	124.6	1.6	6.1	32.8
mBERT	110.2	1.4	5.5	28.4

The computational analysis in Table 8 reveals that CLAS-Net, despite achieving superior accuracy (83% multilingual, compared to mBERT’s 54%), requires additional computational resources. The model’s inference time is 47.6ms per sample compared to mBERT’s 28.4ms—a 68% increase—while memory consumption is 2.1GB versus mBERT’s 1.4GB. These increases stem from the sophisticated components designed for cross-lingual semantic alignment and sentiment feature extraction. The XLM-RoBERTa component contributes substantially to both overhead dimensions: removing it reduces inference time by 51% (from 47.6ms to 23.2ms) and memory by 43% (from 2.1GB to 1.2GB). The BiLSTM-Attention mechanism contributes additional complexity: removing it reduces inference time by 31% (from 47.6ms to 32.8ms) and memory by 24% (from 2.1GB to 1.6GB). The parameter count difference (178.3M vs 110.2M for mBERT) reflects the architectural additions necessary for task-specific sentiment refinement.

Examining the accuracy-efficiency trade-off provides important insights for deployment decisions. CLAS-Net demonstrates a 29 percentage point accuracy improvement while incurring a 68% increase in inference time and 50% increase in memory consumption relative to mBERT. This translates to approximately 0.43% accuracy improvement per 1% additional inference time—a favorable trade-off for applications prioritizing sentiment classification accuracy. However, deployment in real-time scenarios (sub-50ms latency requirements for social media sentiment monitoring or breaking news analysis) requires optimization strategies. For high-volume multilingual data processing (monitoring 1 million news articles daily), dedicated GPU clusters become necessary, though the computational efficiency remains practical for enterprise-scale systems with appropriate infrastructure.

Model compression techniques offer viable paths to reduce computational overhead while preserving accuracy. Knowledge distillation can compress CLAS-Net into lightweight student models achieving 81.5% accuracy with 40-50% parameter reduction (2.1GB to 1.2GB memory), enabling deployment on resource-constrained platforms. Quantization-aware training reduces inference latency to 18.9ms through INT8 precision while maintaining 82.7% accuracy, suitable for latency-critical applications. Combined pruning and quantization strategies achieve 5.7× memory reduction at the cost of 1.2% accuracy, enabling edge device deployment. These optimization techniques enable CLAS-Net deployment in production systems while maintaining both accuracy and computational feasibility.

The experimental setup for efficiency testing consisted of an NVIDIA A100 GPU with 40GB memory and Intel Xeon Platinum 8380 CPU, with batch size set to 32 for all configurations. All measurements represent the average of 1,000 inference passes on the test dataset, excluding the initial warm-up period.

#### 3.2.4 Comparison with state-of-the-art research.

Positioning CLAS-Net within the broader landscape of cross-lingual sentiment analysis research provides important context for evaluating its contributions. The following comparison examines performance improvements achieved by recent approaches and contextualizes the results within the field’s development trajectory (Table 9).

**Table 9:** Recent cross-lingual sentiment analysis models performance improvement comparison.

Research	Evaluation Dataset	Experimental Improvement^*^
CLAS-Net (Ours)	English-Portuguese cross-language dataset	+29%
Hassan et al. [14]	English-Arabic/Spanish Parallel Dataset	+5%
An et al. [15]	English-Chinese Cross-lingual Dataset	+8%
Bhattacharya et al. [16]	RAVDESS+EmoDB Bilingual Dataset	+15.6%
Zehra et al. [19]	Four-language Cross Corpus	+15%

^*^Performance improvements under respective experimental settings

Comparing various research works from a performance improvement perspective reveals significant differences in existing cross-lingual sentiment analysis methods. Hassan et al. [14] achieved a 5% improvement on English-Arabic and Spanish parallel datasets. An et al. [15] achieved an 8% improvement in English-Chinese cross-lingual transfer scenarios. In more complex multilingual scenarios, Bhattacharya et al. [16] and Zehra et al. [19] achieved 15.6% and 15% performance improvements respectively, indicating that model improvement potential increases with the number of languages processed. The CLAS-Net model proposed in this paper achieved a significant 29% improvement on Portuguese Tweets and News Sentiment datasets, thoroughly validating the model’s superiority in multilingual sentiment analysis tasks. Through innovatively combining XLM-RoBERTa’s cross-lingual contrastive learning capability with BiLSTM-Attention’s precise sentiment feature extraction capability, CLAS-Net successfully breaks through the performance limitations of existing methods, providing new research directions for the field’s development.

The choice of combining XLM-RoBERTa with BiLSTM-Attention in CLAS-Net is theoretically motivated by their complementary strengths in addressing key challenges of cross-lingual sentiment analysis. While other models like mBERT or XLM [4] provide baseline cross-lingual capabilities, they lack specialized mechanisms for sentiment-specific feature extraction. XLM-RoBERTa was selected for its superior cross-lingual representation ability through masked language modeling across 100 languages and its robust attention mechanism for contextual understanding. This component effectively bridges the semantic gap between languages through contrastive learning, creating a unified semantic space. The BiLSTM-Attention component, unlike simple feedforward networks used in prior work [14,15], specifically addresses sentiment complexity through bidirectional sequence processing and an attention mechanism that captures long-range dependencies critical for sentiment analysis. This architecture surpasses transformer-only designs [16] by combining global semantic alignment with sentiment-specific feature extraction. The experimental results confirm this theoretical rationale, demonstrating that this unique combination successfully breaks through the performance limitations of existing methods, providing new research directions for the field’s development.

### 3.3 Discussion

The CLAS-Net model demonstrates excellent performance in cross-lingual sentiment analysis through innovative architectural design and experimental validation. The experimental results verify the model’s technical advancement and highlight its practical value in addressing challenges of global information dissemination. This section discusses the implications of the findings across key dimensions.

1. **Comprehensive Performance Validation Across Monolingual and Multilingual Scenarios:** Experimental results demonstrate significant performance improvements in both monolingual and multilingual contexts. Accuracy rates of 92% and 89% were achieved for English and Portuguese respectively, representing 29% improvement over the baseline model, while multilingual tasks maintained 83% accuracy. Through ablation experiments, the necessity of core components was validated, with XLM-RoBERTa removal resulting in approximately 11% accuracy decrease and BiLSTM-Attention removal causing 18% performance degradation. These results confirm the crucial role of contrastive learning in cross-lingual semantic alignment and the model’s rapid convergence characteristics (16 training epochs). The model’s demonstrated effectiveness across diverse linguistic contexts suggests strong potential for addressing large-scale cross-lingual sentiment analysis demands.
2. **Practical Application Value in Real-World Scenarios:** Experimental validation on Portuguese Tweets and News Sentiment datasets demonstrates the model’s adaptability in realistic social media and news environments. CLAS-Net’s accurate sentiment identification (28.7%, 34.0%, and 21.7% for positive, neutral, and negative sentiments respectively) demonstrates substantial practical value against the backdrop where 87.9% of internet users access news through social media. Notably, the analysis of confusion matrices reveals that neutral sentiment classification presents distinguishable characteristics compared to polarized expressions, stemming from the inherent linguistic ambiguity of neutral expressions containing subtle nuances. This capability provides reliable technical support for global public opinion monitoring and cross-cultural communication.
3. **Future Research Directions and Methodological Extensions:**While the model demonstrates strong performance on resource-rich languages including English and Portuguese, the current study focuses on validating the architectural contribution on representative text domains (news and social media). Temporal drift analysis and cross-backbone transferability represent valuable extensions that warrant separate investigation with dedicated resources, as they address deployment stability and framework generalization rather than core architectural innovation. Extending CLAS-Net to low-resource languages through transfer learning, domain adaptation across diverse text genres, and evaluation on code-mixed datasets remains important future work. These extensions will establish whether the principles of sentiment-polarized semantic alignment and sequential sentiment-specific refinement generalize beyond the current evaluation scope, ultimately validating the broader applicability of the proposed framework for global multilingual sentiment analysis.

## 4 Conclusion

The CLAS-Net model achieves significant performance improvements in news sentiment analysis tasks. In single-language (English and Portuguese) scenarios, accuracy rates of 92% and 89% were achieved respectively, representing a 29 percentage point improvement over the baseline model, while multilingual scenarios demonstrated 83% accuracy with identical improvement magnitude. Ablation experiments validate the importance of core components, with the XLM-RoBERTa module effectively extracting cross-lingual semantic features and the BiLSTM-Attention module capturing complex dependency relationships in sentiment semantics. The synergistic effect between these components drives the model’s strong performance. CLAS-Net demonstrates substantial adaptability and practical value in handling real-world social media and news data, providing reliable technical support for cross-lingual public opinion monitoring and analysis. Future research directions include further architectural optimization, extension to related cross-lingual text analysis tasks, and targeted improvements for neutral sentiment classification and data augmentation techniques. Such efforts will establish deeper expansion in fields including AI-assisted decision-making and social sentiment monitoring.

## Appendix: Proofs of Corollaries and Theorems

**Theorem 1** (Cross-lingual Semantic Consistency Theorem). *For any semantically equivalent text pair $\left(x_{s},x_{t}\right)$ in languages* *l*_*s*_ *and* *l*_*t*_, *when the optimization objective ${ℒ}_{\text{total}}$ converges, there exists a constant C > 0 such that the feature representations satisfy:*


$$\mathbb{P}(\|\Phi (x_{s})-\Phi (x_{t}){\|}_{2}\leq \epsilon )\geq 1-\delta \;\text{where}\;\delta \leq C\cdot \exp\left(-\frac{N\epsilon^{2}}{2\sigma^{2}}\left(1-\frac{\tau }{\lambda_{1}}\right)\right)\cdot \;\;{\left(1+\frac{\lambda_{2}}{\lambda_{1}}\cdot E_{p(z^{s},z^{t})}[\text{KL}(p(z^{s}|z^{t})\|q_{\phi }(z^{s}|z^{t}))]\right)}^{-1}\cdot \;\;\exp\left(-\lambda_{4}\cdot \int_{\|z{\|}_{2}\leq R}|{𝒫}_{s}(z)-{𝒫}_{t}(z)|dz\right)$$

*Proof:* First, we begin with the convergence of the optimization objective ${ℒ}_{\text{total}}$. When the objective function converges:


$$\nabla_{\theta }{ℒ}_{\text{total}}=0$$

This implies that for any semantically equivalent pair $\left(x_{s},x_{t}\right)$, their feature representations satisfy:


$$\|\Phi (x_{s})-\Phi (x_{t}){\|}_{2}^{2}\leq \frac{2{ℒ}_{\text{contra}}}{\lambda_{1}}+\frac{2\lambda_{2}}{\lambda_{1}}{ℒ}_{\text{MI}}$$

For the contrastive loss term, we can derive:


$${ℒ}_{\text{contra}}\leq -\log\frac{\exp(-\|\Phi (x_{s})-\Phi (x_{t}){\|}_{2}^{2}/\tau )}{Z}\;=\frac{\|\Phi (x_{s})-\Phi (x_{t}){\|}_{2}^{2}}{\tau }+\log Z$$

where *Z* is the normalization factor. For the mutual information loss:


$${ℒ}_{\text{MI}}=-E_{p(z^{s},z^{t})}[\log q_{\phi }(z^{s}|z^{t})]\;=E_{p(z^{s},z^{t})}[\text{KL}(p(z^{s}|z^{t})\|q_{\phi }(z^{s}|z^{t}))]+H(z^{s}|z^{t})$$

where $H(z^{s}|z^{t})$ is the conditional entropy. Combining with the invariance regularization term:


$$E[\|\Phi (x_{s})-\Phi (x_{t}){\|}_{2}^{2}]\leq \frac{2\sigma^{2}}{N}{\left(1-\frac{\tau }{\lambda_{1}}\right)}^{-1}\;\;\times \log\left(1+\frac{\lambda_{2}}{\lambda_{1}}E[\text{KL}(p\|q_{\phi })]\right)$$

For the distributional difference term, using Pinsker’s inequality:


$$\text{KL}({𝒫}_{s}\|{𝒫}_{t})\geq \frac{1}{2}\|{𝒫}_{s}-{𝒫}_{t}{\|}_{1}^{2}$$

Combining Markov’s inequality and Hoeffding’s inequality, for any ϵ>0:


$$\mathbb{P}(\|\Phi (x_{s})-\Phi (x_{t}){\|}_{2}>\epsilon )\leq \frac{E[\|\Phi (x_{s})-\Phi (x_{t}){\|}_{2}^{2}]}{\epsilon^{2}}\;\leq \exp\left(-\frac{N\epsilon^{2}}{2\sigma^{2}}\left(1-\frac{\tau }{\lambda_{1}}\right)\right)$$

For the feature distributions:


$$\|{𝒫}_{s}-{𝒫}_{t}{\|}_{1}\leq \sqrt{2\text{KL}({𝒫}_{s}\|{𝒫}_{t})}\;\leq \exp\left(-\lambda_{4}\int_{\|z{\|}_{2}\leq R}|{𝒫}_{s}(z)-{𝒫}_{t}(z)|dz\right)$$

Therefore, letting *C* be a sufficiently large constant:


$$\delta \leq C\cdot \exp\left(-\frac{N\epsilon^{2}}{2\sigma^{2}}\left(1-\frac{\tau }{\lambda_{1}}\right)\right)\cdot \;\;{\left(1+\frac{\lambda_{2}}{\lambda_{1}}\cdot E_{p(z^{s},z^{t})}[\text{KL}(p(z^{s}|z^{t})\|q_{\phi }(z^{s}|z^{t}))]\right)}^{-1}\cdot \;\;\exp\left(-\lambda_{4}\cdot \int_{\|z{\|}_{2}\leq R}|{𝒫}_{s}(z)-{𝒫}_{t}(z)|dz\right)$$

Thus, the theorem is proved. In particular, when $\lambda_{1}>\tau$ and $\lambda_{2},\lambda_{4}>0$, the upper bound decays exponentially with the increase in training samples *N*. □

**Corollary 1** (Cross-lingual Sentiment Consistency Guarantee). *Given semantic features* **Z** *from XLM-RoBERTa algorithm satisfying Theorem 1, for any language pair $\left(l_{s},l_{t}\right)$ with equivalent text pair $\left(x_{s},x_{t}\right)$, there exists a constant η>0 such that the sentiment classification probability distributions satisfy:*


$$\|𝒫(y|x_{s})-𝒫(y|x_{t}){\|}_{1}\leq \eta \cdot \Omega (𝐀,𝐒)\;\text{where}\;\Omega (𝐀,𝐒)=\exp\left(-\frac{\lambda_{1}}{L}\sum_{l=1}^{L}\text{tr}({𝐀}^{\left(l\right)}{{𝐀}^{\left(l\right)}}^{T})\right)\cdot \;\;{\left(1+\lambda_{2}\cdot \text{MI}(𝐒,𝐄)\right)}^{-1}\cdot \;\;\sqrt{\frac{1}{N}\sum_{i=1}^{N}\|{𝐒}_{i}-\text{SG}({𝐅}_{i}^{\left(L\right)}){\|}_{2}^{2}}$$

*Proof:* First, according to Theorem 1, for semantically equivalent text pair $\left(x_{s},x_{t}\right)$, their feature representations satisfy:


$$\mathbb{P}(\|{𝐙}_{s}-{𝐙}_{t}{\|}_{2}\leq \epsilon )\geq 1-\delta$$

Through the BiLSTM encoder, for any time step *t*:


$$\left\|{𝐡}_{t}^{s}-{𝐡}_{t}^{t}{\|}_{2}\leq \|{𝐖}_{ih}{\|}_{2}\|{𝐙}_{s}-{𝐙}_{t}{\|}_{2}+\|{𝐖}_{hh}{\|}_{2}\|{𝐡}_{t-1}^{s}-{𝐡}_{t-1}^{t}{\|}_{2}\;\leq C_{1}\epsilon (1+\rho +\rho^{2}+...+\rho^{t-1}\right)$$

where $\rho =\|{𝐖}_{hh}{\|}_{2}$ and *C*_1_ is a constant. For the gated fusion mechanism:


$$\|{𝐑}_{s}-{𝐑}_{t}{\|}_{2}\leq \|{𝐆}_{s}-{𝐆}_{t}{\|}_{2}\|\tanh({𝐖}_{r}{𝐇}_{s}){\|}_{2}\;+\|{𝐆}_{s}{\|}_{2}\|\tanh({𝐖}_{r}{𝐇}_{s})-\tanh({𝐖}_{r}{𝐇}_{t}){\|}_{2}\;+\|1-{𝐆}_{s}{\|}_{2}\|\text{FFN}({𝐙}_{s})-\text{FFN}({𝐙}_{t}){\|}_{2}$$

For the hierarchical attention mechanism, considering layer *l*:


$$\|{𝐀}_{s}^{\left(l\right)}-{𝐀}_{t}^{\left(l\right)}{\|}_{F}\leq \sum_{i=1}^{h}|\alpha_{i}^{\left(l\right)}|\|{\text{head}}_{i}^{\left(l\right)}(s)-{\text{head}}_{i}^{\left(l\right)}(t){\|}_{F}\;+|\beta^{\left(l\right)}|\|\text{PE}({𝐅}_{s}^{\left(l-1\right)})-\text{PE}({𝐅}_{t}^{\left(l-1\right)}){\|}_{F}$$

By induction, we can obtain:


$$\|{𝐅}_{s}^{\left(l\right)}-{𝐅}_{t}^{\left(l\right)}{\|}_{2}\leq (1+C_{2}\|{𝐀}_{s}^{\left(l\right)}-{𝐀}_{t}^{\left(l\right)}{\|}_{F})\|{𝐅}_{s}^{\left(l-1\right)}-{𝐅}_{t}^{\left(l-1\right)}{\|}_{2}\;\leq C_{3}\exp\left(\frac{\lambda_{1}}{L}\sum_{l=1}^{L}\text{tr}({𝐀}^{\left(l\right)}{{𝐀}^{\left(l\right)}}^{T})\right)$$

For the sentiment knowledge injection module, utilizing properties of mutual information:


$$\|{𝐒}_{s}-{𝐒}_{t}{\|}_{2}\leq \gamma \|\text{CrossAttention}({𝐅}_{s}^{\left(L\right)},𝐄)-\text{CrossAttention}({𝐅}_{t}^{\left(L\right)},𝐄){\|}_{2}\;+(1-\gamma )\|\text{InfoNCE}({𝐅}_{s}^{\left(L\right)},𝐄)-\text{InfoNCE}({𝐅}_{t}^{\left(L\right)},𝐄){\|}_{2}\;\leq \frac{C_{4}}{1+\lambda_{2}\cdot \text{MI}(𝐒,𝐄)}$$

For the final classification probability distribution, applying Lipschitz continuity and gradient boundedness:


$$\|𝒫(y|x_{s})-𝒫(y|x_{t}){\|}_{1}\leq L_{f}\|{𝐒}_{s}-{𝐒}_{t}{\|}_{2}\;\leq L_{f}\sqrt{\frac{1}{N}\sum_{i=1}^{N}\|{𝐒}_{i}-\text{SG}({𝐅}_{i}^{\left(L\right)}){\|}_{2}^{2}}$$

Let $\eta =\max\{C_{1},C_{2},C_{3},C_{4},L_{f}\}$, combining the above inequalities:


$$\left\|𝒫(y|x_{s})-𝒫(y|x_{t}){\|}_{1}\leq \eta \cdot \Omega (𝐀,𝐒\right)$$

where Ω(𝐀,𝐒) takes the form given in the corollary. This shows that when $\lambda_{1},\lambda_{2}$ take appropriate values, cross-lingual sentiment analysis consistency can be theoretically guaranteed. □
